# Peak loads vs. cold showers: the impact of existing and emerging hot water controllers on load management

**DOI:** 10.1080/03036758.2023.2286988

**Published:** 2023-12-12

**Authors:** Daniel Bishop, Theo Nankivell, Baxter Williams

**Affiliations:** a Department of Civil and Natural Resources Engineering, University of Canterbury, Christchurch, New Zealand; b Department of Mechanical Engineering, University of Canterbury, Christchurch, New Zealand

**Keywords:** Hot water cylinders, smart control, demand side management, domestic hot water, load management, stochastic control, demand fulfilment

## Abstract

Electric Hot Water Cylinders (HWCs) offer considerable Demand Side Management in Aotearoa New Zealand, which can provide load management and increase integration of renewable electricity. In this work, scenario analyses are conducted to simulate the impact on Low Voltage transformer load and demand fulfilment of four HWC controller types: setpoint (the default in Aotearoa New Zealand), ripple, smart-power, and smart-thermostat. All controllers reduce peak electricity demand by 14-34% from setpoint, where 34% is the maximum possible reduction with hot water control. Unmet demand, which indicates insufficient hot water and can lead to negative outcomes such as cold showers, is increased by 120% and 12-69% by ripple and smart-power control, respectively, and decreased by 7-31% by smart-thermostat control. Average thermal losses are 2.25 kWh/day for the setpoint controller, and between 2.20-2.76 kWh/day for other controllers. Smart-power controllers demonstrate demand deferral, shifting peak electricity loads to shoulder loads, while smart-thermostat controllers demonstrate demand deferral and valley filling, shifting peak loads to times of lowest demand and smoothing load distribution. Overall, smart controllers improve load management performance with little-to-no increase in unmet demand or thermal losses. Thus, smart controllers are a viable option for Demand Side Management in Aotearoa New Zealand.

**Abbreviations and Nomenclature:** DHW: Domestic Hot Water; DLC: Dynamic Load Control; DSM: Demand Side Management; EV: Electric Vehicle; GHG: Green House Gas; HV: High Voltage; HWC: Hot Water Cylinder; LDC: Load Duration Curve; LV: Low Voltage; MV: Medium Voltage; NZD: New Zealand Dollar; PV: PhotoVoltaic; TOU: Time Of Use; UD: Unmet Demand; WTP: Willingness To Pay; A: WTP function coefficient; B: WTP function coefficient; C_p_: specific heat of water [J/kg/K]; C_trans_: cost imposed by the transformer; HW_suff_: ratio of hot water sufficiency; K_loss,h_: thermal losses for cylinder h [W/K]; K_mix_: thermostatic mixing valve factor; m: WTP function coefficient; P_total_: transformer power demand [W]; P_cap_: transformer capacity [W]; P_h_: Household power demand [W].; P_HWC_: heater element power [W]; P_op_: Transformer limit for Type3 controller [W]; Q_DHW_: heat loss from DHW use [W]; Q_loss_: heat loss from standing losses [W]; t_h_: time horizon [s]; T_amb_: ambient temperature [K]; T_HWC_: temperature of the HWC [K]; T_in_: water inlet temperature [K]; T_min_: minimum temperature before fulfilment failure; T_out_: water outlet temperature [K].; T_set_: temperature setpoint of the HWC controller [K]; $\dot{V}$: flow rate of hot water from the HWC [L/s]; V_avail_: available DHW in the HWC [L]; V_DHW_: volume of hot water draw [L]; V_DHW,expected_: expected time weighted demand; V_DHW,expected,max_: maximum expected DHW demand; V_HWC_: volume of the HWC [L]; wf: time weighting function; ρ: density of water [kg/m3].

## Introduction

### Background

Enhanced electrification and uptake of renewable energies, particularly wind and solar generation, present challenges for power systems (Carley et al. 2017; Upton and Snyder 2017; Joskow 2019). Electricity demand is projected to grow and demand complexity is projected to increase, due to power supply intermittency and two-way power-flows, such as from distributed generation and storage (Orion New Zealand 2022; WEL Networks 2022; Wellington Electricity 2022).

Problems will be particularly significant for distribution networks, which are expected to require the largest cost to transition towards the future power system. As power demands and peak power demands grow, distribution infrastructure will need to be upgraded accordingly, incurring significant expense. In Aotearoa New Zealand, power system investment has been projected to require 35 billion NZD per decade until 2050 (Boston Consulting Group 2022). The greatest share of this investment, 21 billion NZD, is for upgrading distribution infrastructure, such as transformers and powerlines.

Low Voltage (LV) networks are the intermediary between residential consumers, regulated to 240 Volts, and the Medium Voltage (MV) network, which is typically 11 kV. A LV network consists of a LV transformer and an arrangement of powerlines and conductors connecting each consumer to a transformer. While the number of consumers connected to an LV transformer in Aotearoa New Zealand can range from 1-350+, a typical LV network comprises 71 households (Watson et al. 2014). LV transformers connect fewer households than MV or High Voltage (HV) transformers, so are subject to decreased load diversity and higher variability (McQueen et al. 2004). Thus, LV transformers are most sensitive to peak loading and changes in household behaviour and are ideal candidates for Demand Side Management (DSM) (Aybar-Mejía et al. 2021).

DSM shifts times of electricity consumption to reduce peak loads, delaying network upgrades and reducing net-present upgrade costs (Williams et al. 2023b). Additionally, DSM can align electricity demand with generation from renewable sources and reduce Greenhouse Gas (GHG) emissions (Groppi et al. 2021). Thus, DSM for LV distribution networks can facilitate the transition to the future power system by increasing the utilisation of renewable generation and reducing distribution network upgrade costs, thus also reducing electricity costs for consumers.

Electric Hot Water Cylinders (HWCs) are ideal candidates for DSM, as their thermal storage capacity means electricity demand can be decoupled from hot water demand without reducing access to hot water (Arteconi et al. 2012; Williams et al. 2023a). Electric HWCs are installed in over 80% of households in Aotearoa New Zealand (Isaacs et al. 2010) and are already used for load management with ‘ripple’ control (Energy Efficiency and Conservation Authority 2020). While electric HWCs are not the most common method of domestic water heating globally, with gas-heated cylinders and continuous-flow water heaters (‘califonts’) more common in many countries, their ubiquity in Aotearoa New Zealand makes them well-suited for DSM (Stephenson et al. 2018).

Ripple control of HWCs is a basic form of load management, where a ‘ripple’ signal is sent through power lines, which disables or re-enables the water-heaters of the connected houses. Ripple control restricts when HWCs can heat and shifts load away from peak demand times. Ripple control is a low-granularity tool for deferring demand of a large group of consumers, as all houses connected to the ripple circuit are controlled together. In Aotearoa New Zealand, ripples are typically sent to an entire MV network (Energy Efficiency and Conservation Authority 2020). As such, ripple control does not consider individual houses’ hot water requirements and can result in unmet demand, such as cold showers, due to insufficient hot water.

Smart power metres (‘Smart-meters’) record time-series power usage and are used for electricity billing (Electricity Authority Te Mana Hiko 2023). Newer smart-meter models have an independent circuit for household HWC Dynamic Load Control. These smart-metres can control HWCs in the same manner as ripple control (i.e. enabling/disabling the water heaters) but for individual households. This higher-granularity control is useful for power system management, as control can be applied with more spatial accuracy and can be used to adapt to spatial power variations, such as from Solar PV and Electric Vehicle charging.

In addition to the above controls, consumers can utilise switches or timers to shift their HWC electricity demand according to Time-of-Use (TOU) electricity charges, which reduce costs to incentivise consumption at off-peak times (Meridian Energy 2022; Genesis Energy 2023). However, peak times for electricity retailers, who buy electricity at highly aggregated levels, do not always coincide with peak times for LV and MV transformers. Thus, load shifting in response to retailer incentives can produce worse outcomes at the distribution level, especially if households override existing power management systems.

While these preceding control methods all directly regulate electricity demand, research has been conducted on smart-thermostat controllers that instead regulate the cylinder temperature, indirectly regulating power demand (Kepplinger et al. 2014; Kepplinger et al. 2015; Jack et al. 2018; Razaq and Jack 2023; Williams et al. 2023a). Smart-thermostat controls for reducing thermal losses are available in commercial cylinders (Rinnai 2022). Additionally, smart-thermostat controls can be used for DSM, by restricting or inducing heating to reduce or increase electricity demand, respectively. HWCs can be preheated to anticipate upcoming hot water demand, reducing peak electricity demand without compromising hot water demand fulfilment (Arteconi et al. 2012). Additionally, by heating HWCs during times of electricity supply from renewable sources, smart-thermostat controllers can facilitate higher renewable generation and reduce GHG emissions (Williams et al. 2023b). Overall, smart-thermostat controls have greater functionality than existing controls but are also more complex. The degree to which this greater functionality translates to better power system outcomes, and thus whether the increase in complexity is justified, is currently unknown.

This paper addresses HWC control strategies, expressed as four controller types reflecting the options described above, and their application for LV network load management. The controller types assessed are: (1) uncontrolled, as the reference case; (2) ripple control, which restricts heating during national peak times; (3) smart-power control, which restricts heating based on LV transformer load and represents what could be achieved with smart-meter based HWC control, and; (4) smart-thermostat control, which restricts and induces HWC heating based on feedback from the LV transformer.

Each controller type is simulated for a year in a representative distribution network and assessed for local LV transformer peak load, transformer load distributions, and fulfilment of hot water demand.

This paper is focussed on LV network management, as this has the greatest demand variability and is thus best suited for DSM. Additionally, this work is relevant to the MV and HV networks, as these networks can also benefit from similar load management strategies. Overall, this paper aims to establish the trade-offs between HWC controller type and complexity, which will overcome knowledge barriers, enhance the uptake of effective load control, and facilitate the affordable transition to a clean energy system.

### Literature review

Aspects of HWC control for DSM have been investigated in Aotearoa New Zealand. Dortans et al. (2018) calculate the aggregate potential for load curtailment using HWCs in Aotearoa New Zealand, based on the total number of electric HWCs and typical peak water heating loads, but do not account for different methods of HWC control or investigate other uses of HWCs for DSM. Razaq and Jack (2023) use a HWC smart controller to align electricity consumption with generation from solar PV in households in Aotearoa New Zealand, but do not assess the impact of the smart controller on distribution network load or compare the smart controller with ripple control or other methods of smart control.

Good et al. (2015) use a smart controller to utilise thermal storage and reduce consumer electricity costs by implementing DSM with time-varying electricity prices. Griffiths and Whitehouse (2017) present a method to reduce peak electricity demand and reduce consumer electricity costs by implementing HWC smart control and conduct a case study with data from six residential consumers. Sonnekalb and Lucia (2019) show how HWCs can adapt to individual human behaviour, which reduces overall energy consumption without compromising DHW fulfilment. Lin et al. (2017) show how consumer energy costs can be reduced with no impact on demand fulfilment, using a genetic algorithm-based optimisation model for a smart HWC controller. Gelažanskas and Gamage (2016) propose a smart controller that uses a machine-learning algorithm to forecast upcoming DHW demand and use these forecasts to optimise HWC temperature to minimise consumer discomfort, increase water heater efficiency, and respond to DSM requests from electricity utilities. Pulkkinen and Louis (2021) propose a smart controller to implement to provide DSM through load balancing with HWCs. Kepplinger et al. (2015) model the implementation of DSM with HWCs, using a pseudo cost function for communication between cylinder controllers and electricity utility companies, which effectively reduces consumer electricity costs and consumption. Kapsalis and Hadellis (2017) demonstrate the use of an optimal scheduling algorithm to use HWCs for DSM, using real-time electricity prices to communicate between electricity retailers and cylinder controllers. Booysen et al. (2019) model the implementation of non-thermostatic control of HWCs using dynamic programming optimisation and show how this control method can reduce energy costs to consumers by reducing heat loss from standing losses.

The effect of DSM on electricity distribution networks is recognised as an important area of research (Anderson et al. 2018; Jack et al. 2018), which has been evaluated for DSM with Electric Vehicles (Clement-Nyns et al. 2011; López et al. 2015; Williams et al. 2023c), air conditioners (Dehghanpour et al. 2016), and other residential electricity loads (Nguyen et al. 2022). However, no research to date has assessed the effects on electricity distribution networks of DSM with HWCs or compared different HWC control types for their impact on peak electricity demand, transformer function, and Domestic Hot Water (DHW) demand fulfilment.

### Contributions

Foremost, this work establishes the relative benefits of a range of HWC controller types and strategies for HWC load management. Secondly, in assessing smart-thermostat controllers for HWCs, a novel method is presented for the decentralised coordination of HWC heating using agents and ‘cost’ functions for the smart-thermostat controllers. The method can be generalised and used for a range of DSM applications, including the coordination of solar PV dispatch, battery operation, and EV charging.

### Outline

This paper follows the following outline. Section 2 describes the simulation methodology and schedule of assessments. Section 3 provides the theoretical background and introduces models for base electricity demand, DHW demand, HWC temperature models, and transformer models. Section 4 presents the results of the analysis. Sections 5 and 6 provide a discussion and conclusion, respectively.

## Methodology

The relative efficacy of different HWC control types for load management are assessed by simulating the use of each control type in a neighbourhood of houses connected to a single LV transformer, representative of an LV network in Aotearoa New Zealand.

**Figure 1.**
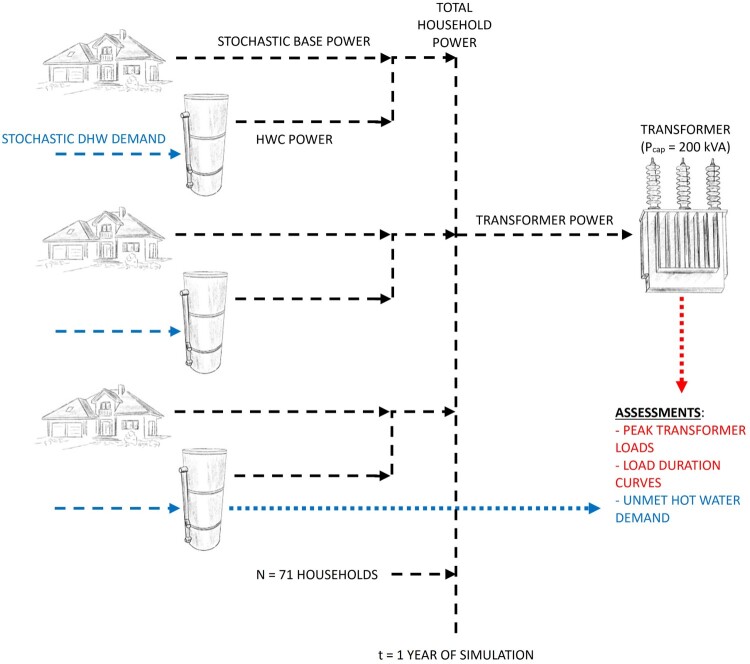
Power-flow diagram of simulated distribution network. Note household base demand and HWC electricity demand are separated for each household.

The distribution network is represented by 71 houses connected to a single 200 kVA-rated transformer (P_cap_ = 200 kW, assuming power factor of 1), depicted graphically in Figure 1. The network is simulated for one year to capture daily and seasonal variations in demand. Each house is attributed a unique base electricity demand, which is household power demand without HWC demand. The number of occupants for the houses are assigned to match the national household size distribution (Statistics New Zealand 2018). Each household is assigned a single HWC and attributed an average daily DHW demand, where HWC size and DHW demand are proportional to household size, based on known plumbing guidelines (HeatingForce 2017 Sep 13) and published data (Basson 1983; Parker et al. 2015), respectively. Simulation input data are presented in the Appendix.

Each household is attributed unique stochastic DHW time-series demand profile. The HWC thermal model calculates cylinder temperatures in response to DHW demand and heating logic, which is dictated by the controller type. The household power demand is calculated as the sum of the household base demand and the HWC power demand. The total electricity load on the transformer is then calculated as the sum of the total power demand for each household. Figure 1 schematically represents the simulated distribution network.

Four controller types reflect the capabilities of existing and emerging technologies and consist of a range of power-based and temperature-based controls, summarised in Table 1.

**Table 1.** Summary of HWC controller types. Power control restricts cylinder heating, analogous to having a switch in-line with the HWC, whereas temperature control involves changing the cylinder temperature setpoint. Fixed temperature control is analogous to thermostats fitted to existing HWCs, where cylinders are heated if the temperature drops below a setpoint.

	Control Type	Power control	Temperature control
(1)	Setpoint (reference)	None	Fixed
(2)	Ripple Control	Ripple	Fixed
(3)	Smart-power control	Smart	Fixed
(4)	Smart-thermostat control	None	Smart

Setpoint control is analogous to inbuilt HWC thermostat control (Williams et al. 2023a), where the cylinder will heat when temperature (T_HWC_) is below the setpoint temperature. Ripple control uses a setpoint controller, but also restricts heating during national peak times, 7am-11am and 5pm-9pm (Powershop 2021). Smart-power uses a setpoint controller and provides feedback between HWC heating and the LV transformer, restricting heating when the transformer reaches 80% of its rated capacity.

For the first three controllers, the temperature setpoint is tuneable but not dynamically controlled. Conversely, smart-thermostat control allows the temperature to vary, allowing the HWC to increase (valley-filling) or decrease (demand deferral) electricity demand when desired. The smart-thermostat controller balances two constraints: fulfilment of DHW demand, and minimisation of transformer electricity demand. The controller logic uses ‘willingness to pay’ (WTP) and ‘cost’ (C_trans_) functions for this purpose. As such, the smart-temperature controller uses a pseudo market-based approach. This market-based approach can reduce the complex task of planning for current and future DHW demand, minimising transformer peak loads, and improving load distributions, by resolving multiple interests into simple costs and transactions. While balancing these considerations with a rules-based approach would require hard coding the specific desired behaviour, the market-based approach codes only the interests of the agents (i.e the HWCs and Transformer) and allows the appropriate behaviour to emerge. As such, this approach allows multiple agents and types of agents to act within the same network, so can be extended to coordinate batteries, electric vehicles, and other infrastructure interests, such as MV and HV transformers.

Market-based approaches have been used for DSM (Torbaghan et al. 2018; Arasteh and Riahy 2019), with varying levels of complexity. The approach used in this work is simple, with each agent considering only a single function and all communication through anonymised price signals. As such, this approach addresses key concerns arising from market-based DSM approaches, such as protection of consumer privacy and providing an efficiently scalable approach (Abedrabboh and Al-Fagih 2023).

Controller logic is summarised as follows:

1. ‘Cylinder on’ **IF** [T_HWC_ < T_set,1_]
2. ‘Cylinder on’ **IF** [T_HWC_ < T_set,2_ & time ≠ (7am-11am OR 5pm-9pm)]
3. ‘Cylinder on’ **IF** [T_HWC_ < T_set,3_ & P_total_ < P_op_]
4. ‘Cylinder on’ **IF** [WTP > C_trans_ & T_HWC_ < T_max_]

where T_set,n_ is the setpoint temperature of controller n, P_total_ is the total transformer load, and P_op_ is the operating power (80% of the transformer’s rated power), and T_max_ is the maximum allowable HWC (80°C).

### Scenarios and assessments

A range of scenarios are simulated, and measures are assessed to cover any trade-offs that could occur, including minimising peak power demands at the cost of increased Unmet Demand (UD), such as cold showers, and to allow fair comparison between controller types. The simulated scenarios are summarised in Table 2. For each scenario all houses utilise a single controller type.

**Table 2.** Scenarios tested in the simulation. Scenarios reflect controller type and how controller inputs are tuned.

Scenarios	Description
1 – Setpoint	T_SP_ = 60°C
2 – Ripple	T_SP_ = 63°C and ‘off’ from 7am-11am & 5pm-9pm
3(A) – Smart-power	T_SP_ = 60°C, P_cap_ = 160kW
3(B) – Smart power	P_cap _= 160 kW and T_SP_ tuned to meet UD of Scenario 1
4(A) – Smart-thermostat	Inputs tuned to minimise UD
4(B) – Smart-thermostat	Inputs tuned maximise load distribution uniformity

Scenarios 1 and 2 establish the outcomes of controllers currently in use, with setpoints of 60°C and 63°C, respectively, to match building surveys (Isaacs et al. 2010). Scenarios 3 and 4 test new smart-power and smart-thermostat controllers controller capabilities. Scenarios 3A&B demonstrate the range of smart-power controller performance, with the minimum setpoint set of 60°C, a minimum temperature to prevent bacterial issues (Ministry of Business Innovation & Employment 2019). Scenario 4A is the smart-thermostat controller tuned to flatten electricity load distribution (i.e. reduce peak demand by valley filling), while attempting to match UD of Scenario 1. Scenario 4B is the smart-thermostat controller tuned to further flatten load distribution while attempting to match UD of Scenario 3A.

For each controller, the following parameters are assessed: (1) Transformer peak demand and annual Load Duration Curve (LDC) [kW]; (2) Unmet DHW demand, assessed as the duration of water use where temperature is under the 50°C [K.min]; and (3) Average daily standing losses per cylinder [kWh/day]. These assessments allow comparison between the different controllers for LV network performance (peak demand and LDC), human factors (unmet DHW demand), and energy use (standing losses).

In addition, an indicative time-series HWC temperature plot and peak day transformer load plot are produced for each controller type, to allow qualitative comparison across controller types.

## Methods

### Household base electricity demand

Base power demand is required for each household in the study. The GreenGrid dataset (Anderson et al. 2018) is used to create annual base power demand profiles. Viable GreenGrid households are limited to those where a full year of data are available and where the hot water circuit was monitored independently. One year of base power demand is calculated for 5 houses which meet these criteria, covering a range of annual electricity energy consumption.

As only five houses from the GreenGrid dataset meet the base demand data requirements, additional base demand profiles are generated from each real household. Each day in the new base-demand profile is attributed the power demand from a random day of the same month of the real household data. Hence, both the daily and seasonal trends are maintained. Each household in the network is attributed a stochastic base power demand profile from one of the 5 source households in equal proportion, as shown in Table A1 in the Appendix.

### HWC physical model

HWC temperature is simulated using a uniform-temperature thermal model, which has been shown to reduce computational time with minimal impact on model accuracy (Kepplinger et al. 2014; Kepplinger et al. 2015; Kapsalis and Hadellis 2017; Pulkkinen and Louis 2021; Williams et al. 2023a). Thermostatic mixing valves regulate outlet temperature, their use is assumed in this work, and output temperature regulated to 50 C. Temperature of water in the HWC is defined in Equation 1, with the heat loss from hot water use defined in Equation 2 and heat loss from standing losses defined in Equation 3. Note in this work, P and Q denote power and heat, respectively, not active and reactive power.

(1) $${\dot{T}}_{\mathrm{HWC}}=\frac{P_{\mathrm{HWC}}\;\text{–}\;Q_{\mathrm{DHW}}\;\text{–}\;Q_{\mathrm{loss}}}{r\;C_{p}\;V_{\mathrm{HWC}}}$$

(2) $$Q_{\mathrm{DHW}}=K_{\mathrm{mix}}\dot{V}C_{p}r(T_{\mathrm{HWC}}\text{–}T_{\mathrm{in}})$$

(3) $$Q_{\mathrm{loss}}=K_{\mathrm{loss}}(T_{\mathrm{HWC}}\;\text{–}\;T_{\mathrm{amb}})$$

where T_HWC_ is the temperature of the HWC [K]; P_HWC_ is the power supplied by the heater element [W]; Q_DHW_ is the heat loss from DHW use [W]; Q_loss_ is the heat loss from standing losses [W]; ρ is the density of water [kg/m3]; Cp is the specific heat of water [J/kg/K]; V_HWC_ is the volume of the HWC [L]; $\dot{V}$ is the flow rate of hot water from the HWC [L/s]; T_in_ is the water inlet temperature [K]; T_amb_ is the temperature of surroundings [K]; K_loss,h_ is an empirically tuned coefficient to a first order approximation of thermal losses for cylinder *h* [W/K]; and K_mix_ is a factor to account for a thermostatic mixing valve, defined in Equation 4.

(4) $$K_{\mathrm{mix}}=\{\begin{array}{c} \frac{T_{\mathrm{out}}-T_{\mathrm{in}}}{T_{\mathrm{HWC}}-T_{\mathrm{in}}},\;\;\;\&\;\;\;T_{\mathrm{HWC}}\geq T_{\mathrm{out}}\\ 1,\;\;\;\&\;\;\;T_{\mathrm{HWC}}<T_{\mathrm{out}} \end{array}$$

where T_out_ is the water outlet temperature [K].

In this analysis, unmet hot water demand (UD) is defined as the product of temperature difference below the minimum temperature (T_min_), and the time of water draw below the minimum temperature, as shown in Equation 5.

(5) $$\mathrm{UD}=\overset{1440}{\underset{0}{\int }}H(\dot{V})H(T_{\min}-T_{\mathrm{HWC}})(T_{\min}-T_{\mathrm{HWC}})\mathrm{dt}$$

where UD has units of Kmins; t_n_ is the time in minutes; and **H** is the Heaviside function (implemented in this case with **H**(0) = 0), which is unitless.

### Low voltage network and transformer physical models

Total electricity demand in the distribution network is defined according to Equation 6.

(6) $$P_{\mathrm{total}}=\overset{h_{n}}{\underset{h\;=\;1}{\sum }}P_{h}$$

where P_total_ is the total electricity demand in the distribution network [W], and P_h_ is the power demand of house *h* [W].

### Stochastic generation of DHW demand

Statistical models are often used to simulate DHW demand profiles. DHWcalc, a programme with statistical models of DHW consumption (Jordan and Vajen 2005), is used in this work to create realistic demand profiles. While other tools are available for generating DHW demand profiles, including Booysen’s (2021) model of DHW demand, and the *LoadProfileGenerator* for residential electricity loads (Pflugradt 2020), DHWcalc is considered the most-used method of generating representative DHW demand profiles (Kepplinger et al. 2014; Braas et al. 2020; Ochs et al. 2020; Pulkkinen and Louis 2021; Williams et al. 2023a).

To simulate representative DHW demand in the distribution network, a unique demand profile is generated from DHWcalc for each house. Average daily DHW demand is set to 50 L per person per day (Basson 1983; Parker et al. 2015), with household occupancy set to match the average for Aotearoa New Zealand, as shown in the Appendix. Two years of data are generated: one year to train the smart-thermostat controller and one year for simulation.

### Smart-thermostat control

The smart-thermostat controller heats the cylinder to balance the need to meet the constraints described in Section 2. This balance is achieved through price functions: the transformer has a cost function that imposes a cost between 0 and 1; the smart-controller has a ‘Willingness-To-Pay’ (WTP) function, which increases in proportion to the need for additional hot water, which also varies between 0 and 1. The cylinder turns on when WTP exceeds transformer cost. The form and limits of the WTP and cost functions mean cylinder heating is totally restricted above the transformer capacity. Hence, HWC heating cannot cause transformer loads above the transformer rated capacity. The parameters used to tune the coefficients are static.

#### Transformer cost function

The transformer generates a cost (C_trans_) linearly proportional to the demand on the transformer, as shown in Equation 7.

(7) $$C_{\mathrm{trans}}=P_{\mathrm{total}}/P_{\mathrm{cap}}$$

where P_total_ is the load on the transformer and P_cap_ is the capacity of the transformer.

This cost function only accounts for loading on the distribution transformer and does not consider load in the wider electricity network. While this work focuses on LV network management, the same principles of peak load reduction apply throughout the electricity system, and the market-based approach presented in this work can be applied at any level, such as to reduce peak loading for MV or HV transformers. Furthermore, the method presented in this work can be extended to include multiple interests at the same time, by combining multiple cost functions, such as from generators and LV, MV, and HV transformers, into a single function to determine the cost for an individual HWC controller.

#### Prediction of DHW demand for the smart-thermostat controller

Expected DHW demand profiles allow the smart-thermostat controller to anticipate upcoming demand and contain the upcoming DHW demand for each minute of the day, weighted to discount demand further in the future. Because future DHW draws have more time for heating in preparation, hot water at the present time is more valuable than potential hot water in the future. The smart-thermostat controller uses a time-weighting function, wf, to discount future demand, meaning it values current DHW needs above potential future DHW needs. Profiles are created for each house from the year of training data, and are generated by simplifying the year of training data into an average day, as follows:

1. For each training year minute, DHW demand over a prediction horizon *t_n_* is convolved with a time-weighting function, wf(t), to calculate the anticipated demand, as shown in Equation 8.
2. Expected demand (V_DHW,expected_) for an average day is determined by calculating the average anticipated demand for each minute of the day.

(8) $$V_{\mathrm{DHW},\mathrm{expected},t}=\overset{t\;+\;t_{h}}{\underset{t}{\sum }}V_{\mathrm{DHW},\mathrm{training}}\left(t)\mathrm{wf}(t\right)$$

where V_DHW,expected_ is the expected upcoming DHW demand of the training set between t and (t + t_h_) in [L]; V_DHW,training_ is the DHW demand from the training set*;* wf(t) is the time-weighing function, shown in Equation 9; and t_h_ is the time horizon, implemented in this case with t_h_ = 24 h, as average DHW demand over greater periods exceeds HWC energy storage capacity and renders longer prediction horizons impractical.

(9) $$\mathrm{wf}(t)=\frac{1}{t_{h}^{n}}\times |(t_{h-t}{)}^{n}|$$

The exponent n changes the relative weighting of future demands, such that higher values of n discount future DHW demands to a greater degree. In this analysis, a value of n = 3 is used, to produce profiles that sufficiently account for upcoming demand.

#### Willingness-to-pay function

The volume of hot water available in the cylinder is a function of cylinder capacity and temperature, defined in Equation 10.

(10) $$V_{\mathrm{avail}}=V_{\mathrm{HWC}}\times \frac{T_{\mathrm{HWC}}-T_{\mathrm{out}}}{T_{\mathrm{out}}-T_{\mathrm{in}}}$$

where $T_{\mathrm{out}}$ is the outlet temperature from the HWC; and $T_{\mathrm{in}}$ is the temperature of cold water entering the HWC.

Hot water sufficiency (HW_suff_) is a ratio expressing how much hot water is available compared to expected demand, expressed in Equation 11.

(11) $$HW_{\mathrm{suff}}=\;(V_{\mathrm{DHW},\mathrm{expected},t}-V_{\mathrm{avail}})/V_{\mathrm{DHW},\mathrm{expected},\max}$$

where $V_{\mathrm{DHW},\mathrm{expected},t}$ is the expected demand at time *t* and $V_{\mathrm{DHW},\mathrm{expected},\max}$ is the largest expected demand in the dataset. The function is formulated such that the function evaluates to 0 when available DHW is equal to expected demand and no additional hot water is required, evaluates to 1 when the deficit of hot water is at a maximum, and is negative when available DHW exceeds expected demand.

The WTP function is shown in Equation 12.

(12) $$\mathrm{WTP}=\{\;\;\begin{array}{c} B+(1\text{-}B)(HW_{\mathrm{suff}}{)}^{m},\;\;HW_{\mathrm{suff}}>0\\ \;\;\;\;\;\;\;\;\;\;\;\;\;\;\;\;\;\;\frac{B(HW_{\mathrm{suff}}+A)}{A},\;\;HW_{\mathrm{suff}}\leq 0 \end{array}$$

where A sets the maximum negative HW_suff_, and hence the maximum overheating; B sets the WTP at HW_suff _= 0; and the m exponent describes the shape of the function where HW_suff_ is positive, when additional hot water is required. Increasing parameters *A* and *B* increases the willingness for the controller to induce heating in response to low transformer costs, which results in valley filling for the transformer. *A* is the HW_suff_ value where cost equal zero, so sets the upper limits for HWC over-heating, and *B* is the cost at HW_suff_ = zero, so indicates the allowable overheating when demands are balanced. Increasing *m* defers HWC heating until the need is large and immediate.

**Figure 2.**
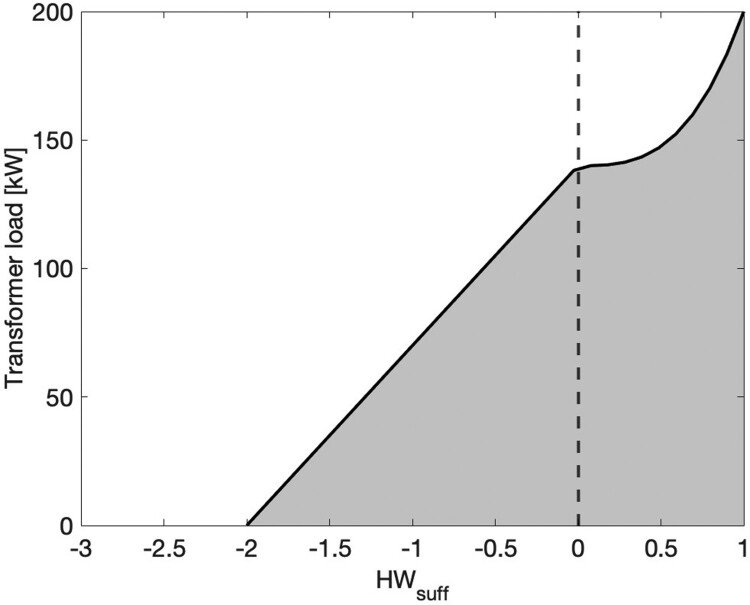
HWC heating behaviour for a range of transformer loads and HW_suff_ values, where the grey shaded area depicts where WTP > C_trans_. This figure is for coefficients: A = 1, B = 0.7, and m = 3.

Given the form of the WTP and C_trans_ functions, cylinder heating is encouraged when DHW is required and discouraged when transformer load is high. However, the cylinder will still heat when DHW supply is sufficient, if the transformer load is low, thus promoting valley filling. HWC heating behaviour is shown in Figure 2, where the shaded area depicts the region where the HWC will heat (WTP > C_trans_).

### Simulation flowchart and summary of assumptions

**Figure 3.**
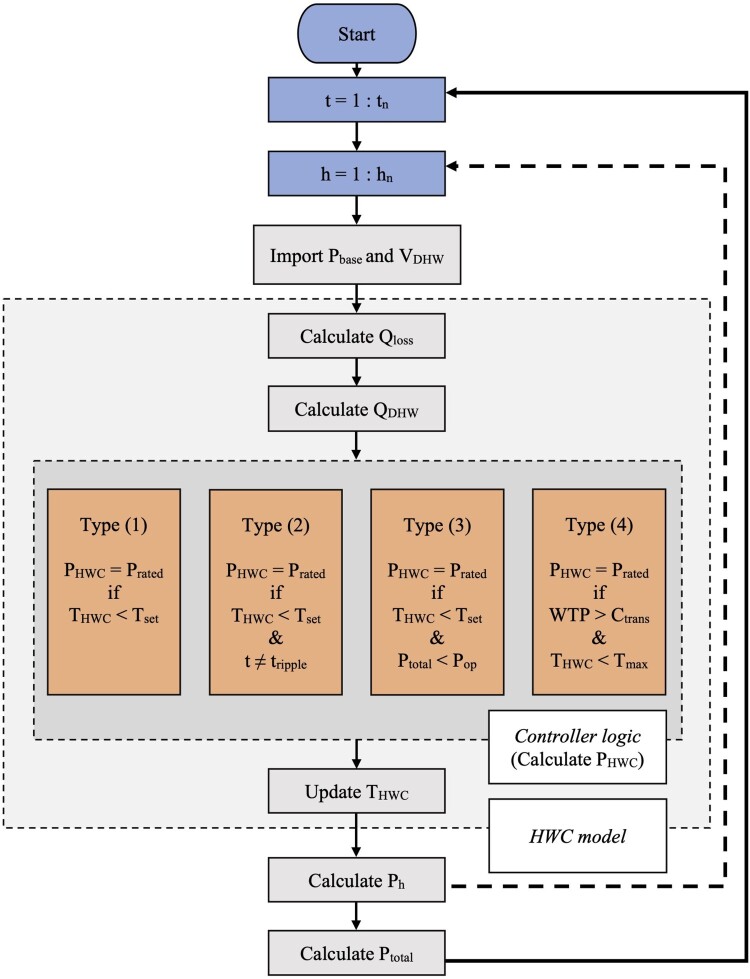
Flowchart showing the order of computation for simulation of households, cylinders, and total electricity demand in the distribution network.

The district is simulated according to the flowchart shown in Figure 3. All inputs to the simulation are summarised in the Appendices (Tables A1-A4).

The following assumptions are used in these analyses:

- Household sizes match national distribution.
- One HWC per household, with capacity to match plumbing guidelines.
- All HWC heating capacities equal 2.3 kW.
- DHW consumption of 50 L per person per day.
- HWCsutilise a fully mixed, uniform-temperature model.
- Power factor assumed to be 1.
- Conductor and transformer resistive losses assumed to be zero.

## Results

### Load duration curves (LDCs) and unmet demands

**Figure 4.**
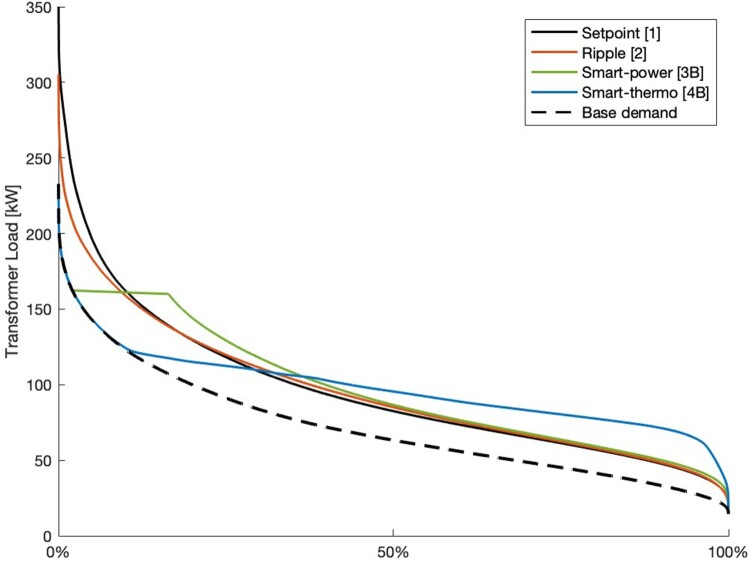
Load Duration Curves of total transformer load for the simulated year for base demand without HWC power, and for total demand with each controller type.

**Figure 5.**
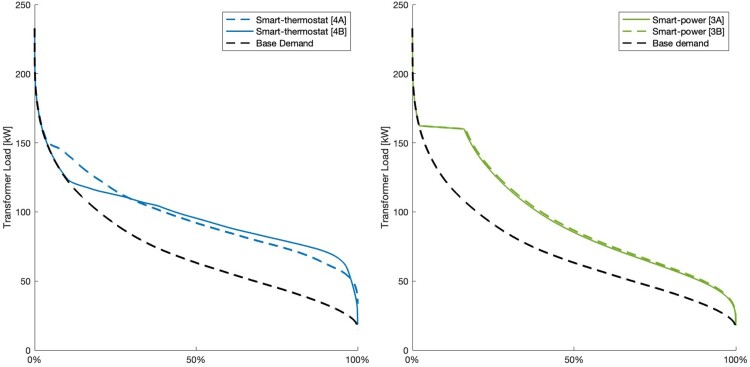
Load Duration Curves for both versions of the smart-thermostat (left) and smart-power (right) controllers.

Load Duration Curves (LDCs) for each controller type are presented in Figure 4, showing how the smart-power and smart-thermostat controllers can reduce peak loads. Load duration curves for both versions of the smart-power and smart-thermostat controllers are shown in Figure 5, which illustrates how the smart-thermostat controller can further reduce peak loads and flatten the LDC, compared to the smart-power controller.

Transformer loads for the 0th, 10th, 50th, and 90th percentile for each controller type and the relative difference from Scenario 1 are shown in Table 3, which illustrates the ‘flattening’ effect of the smart controllers, compared to the ripple and setpoint controllers.

**Table 3.** Total transformer load for the simulated year for each of the scenarios and the corresponding relative changes.

Scenario	Percentile transformer load (kW)				Percentile transformer load change from Scenario 1 (%)			
	0	10	50	90	0	10	50	90
1	354	162	82	48	0%	0%	0%	0%
2	305	159	85	48	−14%	−2%	+4%	0%
3A	233	161	88	51	−34%	−1%	+7%	+6%
3B	233	161	87	50	−34%	−1%	+6%	+4%
4A	233	142	92	63	−34%	−12%	+12%	+31%
4B	233	124	95	71	−34%	−23%	+16%	+48%

**Figure 6.**
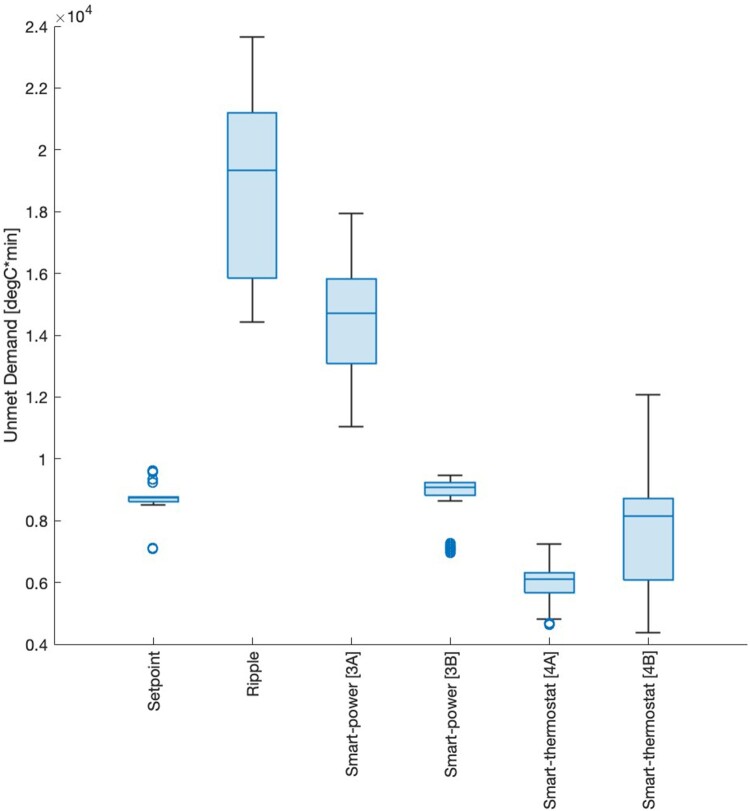
Box plots of Unmet Demand for each simulated scenario.

Box plots of annual Unmet Demands for each controller are presented in Figure 6, which shows the peak load reductions of the smart controllers can be achieved without increased Unmet Demand.

### Cylinder temperatures and transformer loads

**Figure 7.**
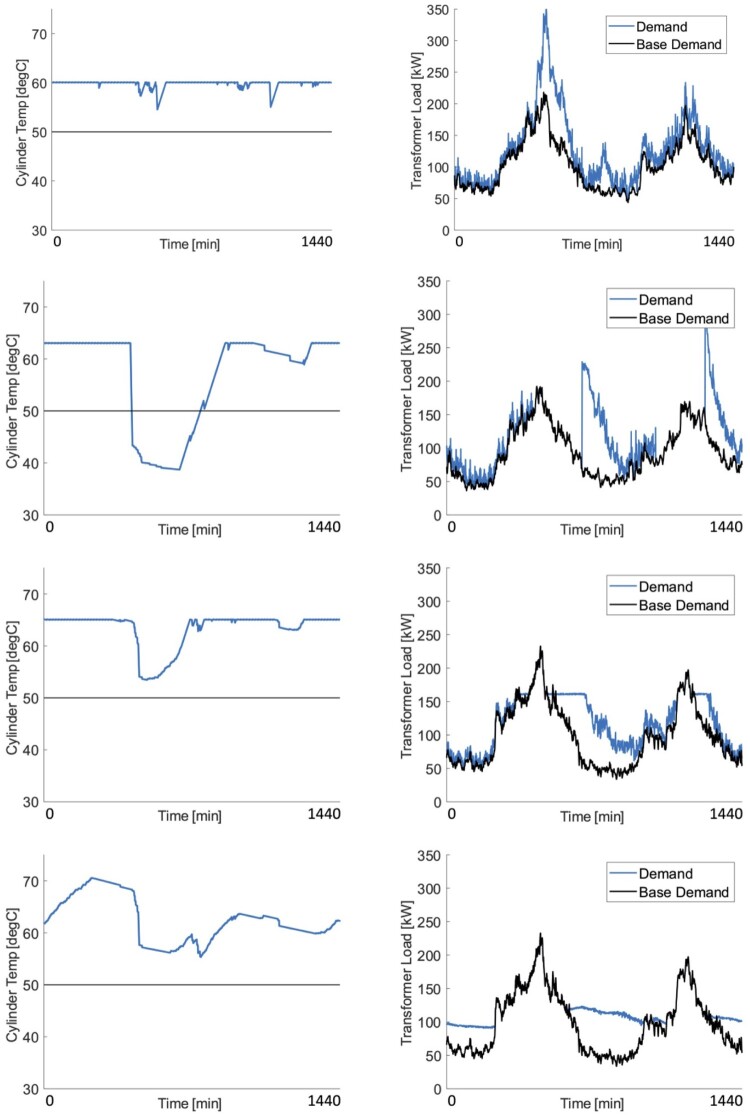
Cylinder temperature profiles for a single HWC (left) and transformer load profiles (right), for each controller type on the peak day of transformer load (which varies between controllers), where time ranges from midnight to midnight and the horizontal line at 50 °C shows the minimum temperature required to prevent unmet demand. Controller types top-to-bottom: Type 1, Type 2, Type 3B, Type 4B.

Cylinder temperature profiles and transformer load profiles for the peak load day are shown in Figure 7. The maximum allowable temperature of 80 °C, which is not met by any of the cylinders. Note results are not all presented for the same day, as the peak day varies between different controllers.

### Summary of results and standing losses

A summary of key results for each scenario, including controller input parameters, relative peak load reduction from Scenario 1, median Unmet Demand, and average standing losses, is shown in Table 4.

**Table 4.** Summary of key results for each scenario**.**

Scenario	Parameters	Peak transformer load reduction (% from S1)	Median UD (×10^3^ °Cmin)	Average Standing losses per cylinder (kWh/day)
1	Setpoint = 60°C	0%	8.7	2.25
2	Setpoint = 63°C	14%	19.3	2.22
3A	Setpoint = 60°C; P_cap _= 160kW	34%	14.7	2.20
3B	Setpoint = 65°C; P_cap _= 160 kW	34%	9.1	2.47
4A	A = 1; B = 0.6; n = 1.2	34%	6.1	2.56
4B	A = 2.7; B = 0.52; n = 2.2	34%	8.1	2.76

## Discussion

### Key results and controller performance

All controller types reduce peak demand between 14 - 34% from the setpoint controller, as shown in Table 4. At a 34% reduction, all peak demand is from base demand, so no further peak demand decreases are possible from HWC control. As ripple control only results in a 14% decrease in peak demand, HWC heating still contributes to LV peak loading, demonstrating the limited efficacy of ripple control. In addition to peak demand reductions, smart controllers affect load distribution, as shown in Figures 4 and 5. Smart-power control reduces peak demands through demand deferral, shifting peaks to ‘shoulders’ (times of off-peak demand where demand is still high), whereas smart-thermostat controllers perform both demand deferral and valley filling, resulting in a more uniform LDC. Setpoint tuning for the smart-power controller has little impact on LDC, whereas tuning the coefficients in the smart-thermostat controller can significantly shift LDC.

The impact of each controller on demand fulfilment is shown in Figure 6. Setpoint and Ripple controllers establish the bounds of UD, between 8,700–19,300 degree-minutes annually, where peak demand reductions are generally associated with increased UD. Smart controllers offer better load management and hot water fulfilment outcomes compared to ripple, and thus are superior controller types. Additionally, the smart-thermostat controller shows the highest load management and lowest UD, due to anticipating DHW consumption. Hence, the smart-thermostat presents the most optimal controller for the trade-off between transformer and DHW outcomes. Additionally, smart-thermostat parameter tuning does not result in better load management and only increases UD, so the smart-controller is likely close to its optimal configuration and limitations on further improvements are due to seasonal variation in DHW demand and base electricity demand. Finally, the spread of UD for controller scenarios 3A and 4B indicates controller parameters should be tuned at the household level to maximise outcomes for each household.

Average HWC thermal losses are summarised in Table 4 and range between 2.20–2.76 kWh per day. The smart-power controller has little-to-moderate impact on standing losses, between −1–11.3% from the setpoint. The smart-thermostat controller produces the highest losses between, 0.36-0.56 kWh (16.2-25.2%) above the best performing smart-power controller. Overall, the difference in standing losses is minimal and can be seen as a worthwhile trade-off considering the load management potential, which can be addressed by increased HWC insulation.

#### Summary and takeaways

- All controllers reduce peak loads beyond the setpoint controller.
- Smart controllers can shift residential load distribution. Smart-power controllers defer demand, and smart-thermostat controllers both defer demand and valley-fill.
- Smart-thermostat controllers have the additional capability of heating in response other signals, such as low electricity costs, and can therefore facilitate the integration of renewable generation.
- Generally, peak-load reduction results in increased UD. An exception is the smart-thermostat controller, which can reduce peak load and UD.
- Ripple and setpoint controllers, given the ubiquity, indicate a likely acceptable range of demand fulfilment. All smart controllers demonstrate significant load management within this range of UD. Thus, the smart-thermostat controller should be acceptable to New Zealand consumers.
- Smart controllers cause a small increase in standing losses, which can be addressed with added HWC insulation.

### Practical implications and demand side management potential

Hot water control is emerging as a viable option for Demand Side Management in Aotearoa New Zealand, given its potential as a low-cost means of reducing peak electricity loads, deferring infrastructure upgrades and reducing consumer electricity costs. Many controller options exist, and the decisions made now regarding controller upgrades will have an impact well into the future. This work details the relative efficacy of different controller types and the implications for load management and DHW demand fulfilment. As such, this work addresses a key current issue and is well placed to inform electricity companies and policy makers about the implications of different controller technologies and the magnitude of potential benefits.

Smart-power controllers are currently commercially available (Octopus Energy 2023) and, as such, are familiar to grid operators. Conversely, smart-thermostat controllers, while demonstrating greater benefits, are still largely in the research stage. Additionally, smart-thermostat controllers would need to be retrofitted to a cylinder and are akin to a commercial electronic device, rather than a traditional method of electricity load management. Thus, smart-thermostat controllers are less familiar to grid operators, which can introduce a barrier to adoption.

In addition to controller uptake, utilising the full potential of these technologies requires real-time monitoring of grid assets, such as transformers, to allow dynamic responses. This monitoring requires coordination between electricity retailers, grid operators, and consumers, although much of the required coordination can be automated, as demonstrated by the market-based control approach implemented in this work.

All smart controllers have input parameters that can be tuned. Controller inputs can be tuned to reflect individual preference for electricity costs and hot water fulfilments, and other interests, such as preferred distribution curves. Thus, consumers and electricity companies can tune the parameters of their cylinders and transformers, respectively, to produce their desired peak demands and levels of UD.

To be economically feasible, the aggregate costs for load controllers for a district, including installation, should be at least lower than the cost required to upgrade the transformer. Smart controllers are an emerging technology, and the likely cost for such controllers, considering existing smart switches on the commercial market, is in the order of 100 NZD. The technical capacities of these existing devices are similar to the requirements for smart-power and smart-thermostat controllers. Hence, costs for these controllers are likely to be similar. As a transformer replacement may cost ∼100,000 NZD, depending on its size and design, the aggregate cost of load controllers is likely to be lower than the infrastructure upgrade costs for a network without load control. For example, if a $100,000 transformer upgrade is considered, each HWC controller would need to cost less than ∼1,400 NZD to have greater economic feasibly than the transformer upgrade. Additionally, some of the cost of these controllers may be borne by consumers, as time-of-use power rates provide a direct economic incentive to the household.

### Limitations and future work

This work specifically investigates load management in Low Voltage networks, as these have the least diversity and highest variability, and thus are most amenable to Demand Side Management. However, other constraints were ignored, such as MV and HV transformer load management, electricity price, and local renewable generation. Even without these considerations, this work accurately indicates the relative performance of controllers, displaying their capabilities and mechanisms of action. Future work can add these constraints and assess HWC control across all use-cases.

The HWC thermal model in this work is a uniform temperature, also called ‘fully mixed’, model. The use of the fully mixed model is well-established and justified in the literature. However, in general, all simple thermal models, mixed or stratified, will introduce error, as real HWCs transition between different modes at different times, due to the mixing and stratifying effects of heating, standing, and draw-offs. Thus, simplified models are less accurate than more complex thermal models. However, the uniform temperature model has been shown to be a viable method of reducing computation time with minimal increases in error (Kepplinger et al. 2014; Kepplinger et al. 2015; Kapsalis and Hadellis 2017; Pulkkinen and Louis 2021).

Additionally, as the uniform temperature model neglects stratification, each DHW draw produces a temperature drop at the location of the temperature sensor, which can cause the controller to initiate heating. In non-uniform conditions, a time delay exists between DHW draws and temperature drops at the temperature sensor, so cylinder heating is delayed from water use. In aggregate, delayed heating demands could act to reduce peak transformer demands. Thus, the uniform temperature model may overestimate peak loading for the setpoint controller. A dynamic multi-nodal thermal model accounting for thermal mixing could improve the accuracy of power demand predictions by accounting for inherent heating deferral due to the transient thermal response of the physical system.

Parameter sweeps are not conducted for each controller. Some controllers have multiple inputs, such as the temperature setpoint and transformer load cut-off for the smart-power controller. To simplify the analysis, these inputs are manually tuned to meet a standard unmet demand and provide a basis for meaningful comparisons between controllers. However, non-linear relationships exist between controller input parameters, so controllers may perform optimally and have highly desirable traits at a specific set of these coefficients. This approach does not provide a full picture of the performance of each controller type, and instead shows the performance of each controller at specific levels of unmet demand. While this approach does not deliver a full analysis of each controller type, it does inform the relative performance of each controller under the practical operating conditions. Thus, this analysis methodology is well suited for the research questions addressed in this work.

A further limitation is the use of the same input parameters for each cylinder in these simulations. While HWC controller parameters can be tuned for each household, this is impractical at the scale of the analysis, given the added complexity this addition would introduce. Instead, where possible, controller performance is expressed as a distribution of results or, where impossible, median results are used as the basis of comparison.

Given the need to compare controllers across a consistent basis, controller parameters are tuned to produce certain levels of UD. UD levels are established based on existing controller types and surveys of setpoints, so it is assumed these values provide reasonable bounds for acceptable UD levels. However, a large range exists between these UD bounds. Given the strong relationship between UD and power system outcomes, establishing accepted UD levels would be highly impactful for determining optimal controller operating parameters, and thus a useful contribution for selecting controller types.

In this work, load distribution curves and peak loads are used to assess the impacts of HWC controllers on distribution transformers, and transformer thermal performance is not assessed. The impacts of electrical load on transformer thermal performance are an important consideration for electricity distribution companies, as higher operating temperatures can reduce transformer lifetime. Transformer temperature increases with increased electrical load (Susa et al. 2005), so the load distribution curves presented in this work can be used to estimate transformer thermal performance. However, in applications where thermal performance is a critical consideration, further work may be required to explicitly investigate this.

Additionally, the representative LV network is used in these analyses to determine the number of houses and the size of the distribution transformer, but the network is not modelled in detail. As such, this work does not include analysis of Voltage profiles or energy losses from conduction within the LV network. While this work’s analysis of peak demand and LDCs is sufficient for general comparison of these controllers, implementation of these controllers in detailed network model may be required by electricity distribution companies.

Ripple control is typically applied at the MV level in Aotearoa New Zealand, which is outside the scope of these LV network analyses. Thus, a simplified model of ripple control is used in this work, which restricts heating during times of typical peak demand, representing the most common use case for ripple control. This simplified model limits complexity and allows investigation of ripple control without simulating an entire MV network. As such, the results of this simplified model should be considered indicative of the likely effects of ripple control.

As well as addressing these limitations, future work should include expansion of the market-based model to consider the interaction and coordination between multiple applications, including local generation of solar PV, battery storage, EV charging, and influences such as power price. Multiple agents such as EV charging, battery storage, and HWC heating have inherent control and flexibility, so the market-based approach used in this work is well-suited to coordinating the performance of these different agents.

## Conclusion

Hot water control is a viable option for electricity load management in Aotearoa New Zealand, given its potential as a low-cost to reduce peak demand, defer infrastructure upgrades, and lower consumer power costs. This research tests the benefits of existing and emerging HWC controller types, including ripple, smart-power, and smart-thermostat control. All controller types reduce peak demand from the default setpoint controller. While peak demand reductions generally reduce DHW demand fulfilment, smart controllers offer improved peak power reduction for smaller increases in UD than ripple control, and the smart-thermostat controller offers substantial peak demand reductions while reducing UD. The smart-power controller demonstrates demand deferral, shifting peak loads to shoulder loads. The smart-thermostat controller demonstrates both demand deferral and valley-filling, leading to a more even overall distribution of electricity demand. In all cases, increases in standing loses from the default setpoint controller are minimal. Overall, smart HWC control is a viable and readily implementable solution for power management without substantial reduction in hot water demand fulfilment. Furthermore, the smart-thermostat controller offers the ability to further integrate renewable electricity generation and reduce the greenhouse gas emissions of Aotearoa New Zealand’s power system.

## Appendices

**Table A1.** household base power demand profiles.

House	Proportion	Annual demand (kWh)
1	20%	12398
2	20%	6952
3	20%	12490
4	20%	7134
5	20%	4687
Average annual base demand (kWh)		8732

**Table A2.** Thermal model general inputs.

Parameter	Value	Reference
Cold water inlet temperature (T_in_)	15 °C	(Bulleid 2019 Apr 18)
Minimum DHW temperature (T_min_)	50 °C	(Ministry of Business Innovation & Employment 2019)
Minimum setpoint temperature (T_set_)	60 °C	(Isaacs et al. 2010)
HWC power rating (P_HWC_)	2.3 kW	(Isaacs et al. 2010)
Maximum HWC temperature (T_max_)	80 °C	(Ministry of Business Innovation & Employment 2019)
Ambient Temperature (T_amb_)	18.1 °C	(Isaacs et al. 2010)
Loss Factor (K_loss_)	0.000012V_HWC_ + 0.00043 [kW/°K]	

**Table A3.** Household distribution, HWC capacities, and DHW demand.

Number of occupants	HWC capacity	Proportion of households	Average daily DHW demand
1	50L	8.3%	50L
2	100L	16.6%	100L
3	150L	41.6%	150L
4	200L	25%	200L
5+	250L	8.3%	250L

**Table A4.** Summary of controller input parameters and constraints.

Scenarios		
1 – Setpoint	Setpoint = 60 °C	
2 – Ripple	Setpoint = 63 °C	off from 7am-11am & 5pm-9pm
3(A): – Smart-power	Setpoint = 60 °C	P_op_ = 160 kW (80% of capacity)
3(B) – Smart power	Setpoint = 65 °C	P_op_ = 160 kW (80% of capacity)
4(A) – Smart-thermostat	A = 1; B = 0.6; n = 1.2	
4(B) – smart-thermostat	A = 2.7; B = 0.52; n = 2.2	
